# Impacts of vascular comorbidities on free flap perfusion in microvascular head and neck reconstruction

**DOI:** 10.1007/s00405-023-07913-1

**Published:** 2023-03-10

**Authors:** Mark Ooms, Marius Heitzer, Philipp Winnand, Anna Bock, Marie Katz, Johannes Bickenbach, Frank Hölzle, Ali Modabber

**Affiliations:** 1 Department of Oral and Maxillofacial Surgery, University Hospital RWTH Aachen, Pauwelsstraße 30, 52074 Aachen, Germany; 2 Department of Intensive Care Medicine, University Hospital RWTH Aachen, Pauwelsstraße 30, 52074 Aachen, Germany

**Keywords:** Free tissue flaps, Reconstructive surgical procedures, Hypertension, Diabetes, Atherosclerotic disease

## Abstract

**Purpose:**

Arterial hypertension (AHTN), type 2 diabetes mellitus (DM), and atherosclerotic vascular disease (ASVD) are common vascular comorbidities in patients undergoing reconstruction of the head and neck region with a microvascular free flap. These conditions may affect flap perfusion (microvascular blood flow and tissue oxygenation), which is a prerequisite for flap survival and thus reconstruction success. This study aimed to investigate the impacts of AHTN, DM, and ASVD on flap perfusion.

**Methods:**

Data from 308 patients who underwent successful reconstruction of the head and neck region with radial free forearm flaps, anterolateral thigh flaps, or fibula free flaps between 2011 and 2020 were retrospectively analyzed. Flap perfusion was measured intraoperatively and postoperatively with the O2C tissue oxygen analysis system. Flap blood flow, hemoglobin concentration, and hemoglobin oxygen saturation were compared between patients with and without AHTN, DM, and ASVD.

**Results:**

Intraoperative hemoglobin oxygen saturation and postoperative blood flow were lower in patients with ASVD than in patients without ASVD (63.3% vs. 69.5%, *p* = 0.046; 67.5 arbitrary units [AU] vs. 85.0 AU, *p* = 0.036; respectively). These differences did not persist in the multivariable analysis (all *p* > 0.05). No difference was found in intraoperative or postoperative blood flow or hemoglobin oxygen saturation between patients with and without AHTN or DM (all *p* > 0.05).

**Conclusion:**

Perfusion of microvascular free flaps used for head and neck reconstruction is not impaired in patients with AHTN, DM, or ASVD. Unrestricted flap perfusion may contribute to the observed successful use of microvascular free flaps in patients with these comorbidities.

## Introduction

Reconstruction of the head and neck region with microvascular free flaps after ablative surgery or trauma is commonly performed in patients with comorbidities, including vascular comorbidities such as arterial hypertension (AHTN), type 2 diabetes mellitus (DM), and atherosclerotic vascular disease (ASVD) [1–4].

These vascular comorbidities or their related vascular changes are associated with impairment of systemic macrovascular and microvascular perfusion and therefore may also have an impact on the perfusion of microvascular free flaps [5–9]. AHTN and DM are associated with vascular changes such as endothelial dysfunction (with vasoconstriction), vascular fibrosis (with increased vessel stiffness), and capillary scarcity, leading to impaired macrovascular and microvascular perfusion [6–8]. In addition, ASVD is associated with vascular changes such as vascular plaque formation, vessel wall thickening, and progressive vessel lumen obstruction, leading to impaired macrovascular perfusion [5, 9].

Microvascular free flap perfusion (microvascular blood flow and tissue oxygenation) is a prerequisite for flap survival, and decreased flap perfusion has been shown to lead to microvascular free flap necrosis and failure [10–14]. Despite the potential impairment of microvascular free flap perfusion by AHTN, DM, and ASVD, several studies have found no associations between these comorbidities and flap failure in terms of a difference in flap failure rates between patients with and without AHTN, DM, or ASVD [2–4, 15, 16].

However, to date, no studies have explicitly examined the impacts of these comorbidities or their related vascular changes on microvascular free flap perfusion. Whether AHTN, DM, and ASVD or their related vascular changes affect microvascular free flap perfusion remains unknown in the perspective of getting insights into the physiology of microvascular free flap perfusion and finding an explanation for similar flap failure rates between patients with and without vascular comorbidities.

This study aimed to evaluate the impacts of vascular comorbidities such as AHTN, DM, and ASVD on microvascular free flap perfusion.

## Materials and methods

### Study population

The local ethics committee of the Medical Faculty of RWTH Aachen University approved this retrospective study (EK 309-20). Datasets from a total of 308 patients treated for malignant or nonmalignant disease were used. All patients had undergone microvascular head and neck reconstruction in our Department of Oral and Maxillofacial Surgery between 2011 and 2020. The microvascular free flaps used were radial free forearm flaps (RFFF), anterolateral thigh flaps (ALTF), or fibula free flaps (FFF). Exclusion criteria for the study were flap failure with flap removal, intraoperative or postoperative flap revision, age less than 18 years, diabetes mellitus other than type 2, and missing or incomplete data records.

Clinical records, operation reports, and perfusion measurement protocols were used to obtain data for further analysis. AHTN and DM were diagnosed in accordance with discipline-specific guidelines. ASVD was diagnosed if coronary heart disease, cerebrovascular disease, and or peripheral arterial disease were diagnosed in accordance with discipline-specific guidelines [8, 9]. Smoking status was designated as positive if the patients had actually or in the past smoked on a daily basis for a period of at least 6 months [17]. Prior neck dissection or irradiation status was designated as positive if patients had undergone neck dissection, including an anatomic dissection of the recipient vessel, or neck irradiation, including the area of the recipient vessel, prior to actual surgery. Duration of surgery and flap ischemia was calculated as the time interval between the first incision and the last suture and between flap perfusion cessation on the donor site and flap perfusion onset after anastomosis release on the recipient side, respectively.

All microvascular free flap reconstructions were performed under general anesthesia, and the flaps pedicles were connected to the cervical vasculature as follows: end-to-end configuration for the arterial vessels and end-to-end or end-to-side configuration for the venous vessels. Postoperatively, all patients stayed in the intensive care unit with invasive mechanical ventilation and analgosedation at least until the morning of the first postoperative day. In addition, blood pressure was monitored with an invasive arterial catheter and regulated by continuous central venous vasopressor administration (norepinephrine) with a syringe pump as needed (with a target systolic pressure above 125 mmHg). Heparin (5000 IU) was injected intracutaneously three times daily for 7 days.

### Measurement of perfusion parameters

Measurement of flap perfusion was performed using the O2C tissue oxygen analysis system (O2C Oxygen-to-see, LEA Medizintechnik, Giesen, Germany) described previously [11, 13, 18]. Blood flow (arbitrary units [AU]) was calculated using laser Doppler spectroscopy (830 nm; 30 mW), and hemoglobin concentration (AU) and hemoglobin oxygen saturation (%) were calculated using white light spectroscopy (500–800 nm; 50 W) [10, 11]. Measurements were performed intraoperatively (immediately after anastomosis release) and postoperatively (on the first postoperative morning), with the probe sealed in a sterile cover and placed centrally on the dried skin portion of the flap and a measurement time of 10 s (with a lead time of 4 s) under ambient light compensation control. Flap perfusion parameters, i.e., flap blood flow, hemoglobin concentration, and hemoglobin oxygen saturation, were determined as mean values from 2-mm and 8-mm tissue depth.

### Statistical analysis

For statistical analysis, patients were divided into those with and without AHTN, DM, or ASVD. In addition, patients with AHTN, DM, and or ASVD were pooled as one group to be compared with patients without these comorbidities. They were also divided into those with and without an American Society of Anesthesiologists score (ASA) above 2. Differences between groups were analyzed as follows: for clinical parameters, the chi-squared test, Fisher’s exact test, or Freeman-Halton test for categorical data and the Mann–Whitney test for metric data were used; for flap perfusion parameters, i.e., blood flow, hemoglobin concentration, and hemoglobin oxygen saturation, the Mann–Whitney test for univariable analysis and multiple linear regression models for multivariable analysis were used. For multivariable comparison of patients with and without ASVD, adjustment was made for sex, age, ASA (≤ 2 vs. > 2), prior neck irradiation, flap type (RFFF vs. ALTF vs. FFF), flap location (intraoral vs. extraoral), mean arterial blood pressure (mmHg), and administered catecholamine dose (µg/min/kg). For multivariable comparison of patients with and without DM, adjustment was made for age, body mass index (BMI) (≤ 25 kg/m^2^ vs. > 25 kg/m^2^), flap type (RFFF vs. ALTF vs. FFF), flap location (intraoral vs. extraoral), mean arterial blood pressure (mmHg), and administered catecholamine dose (µg/min per kg body weight). Statistical significance was considered at *p* values < 0.05. For statistical analysis SPSS version 28 (SPSS, IBM, New York, USA) was used.

## Results

### Comparison of clinical parameters between groups

The study population included a total of 308 patients (112 patients with AHTN and 196 patients without AHTN, 40 patients with DM and 268 patients without DM, 42 patients with ASVD and 266 patients without ASVD, and 157 patients with AHTN, DM and or ASVD and 151 patients without AHTN, DM and or ASVD) (Table 1). Patients with and without AHTN or DM differed with respect to age (*p* < 0.001 and *p* = 0.005, respectively) and BMI (*p* < 0.001 and *p* < 0.001, respectively). Patients with and without ASVD differed in sex (*p* = 0.001), age (*p* = 0.030), ASA (*p* = 0.007), and prior neck irradiation (*p* = 0.037). Patients with and without AHTN, DM and or ASVD differed with respect to age (*p* < 0.001) and BMI (*p* < 0.001).

**Table 1:** Clinical parameters of the study population

Variable	All (*n* = 308)	AHTN (*n* = 112)	DM (*n* = 40)	ASVD (*n* = 42)	AHTN/DM/ASVD (*n* = 151)
Sex (n)***					
Male	163 (52.9%)	58 (51.8%)	26 (65.0%)	32 (76.2%)	87 (57.6%)
Female	145 (47.1%)	54 (48.2%)	14 (35.0%)	10 (23.8%)	64 (42.4%)
Age (years)*,**,***,****	64.0 (18.0)	67.0 (17.0)	69.0 (15.0)	66.5 (18.0)	67.0 (17.0)
*BMI (kg/m*^*2*^)*,**,****	24.3 (6.5)	25.9 (6.6)	27.8 (7.2)	25.2 (6.9)	25.9 (6.7)
ASA (n)***					
1 + 2	162 (52.6%)	54 (48.2%)	17 (42.5%)	14 (33.3%)	71 (47.0%)
3 + 4	146 (47.4%)	58 (51.8%)	23 (57.5%)	28 (66.7%)	80 (53.0%)
*Flap type (n)*					
RFFF	151 (49.0%)	60 (53.6%)	19 (47.5%)	18 (42.9%)	78 (51.7%)
ALTF	122 (39.6%)	40 (35.7%)	19 (47.5%)	20 (47.6%)	60 (39.7%)
FFF	35 (11.4%)	12 (10.7%)	2 (5.0%)	4 (9.5%)	13 (8.6%)
*Flap location (n)*					
Tongue	45 (14.6%)	16 (14.3%)	7 (17.5%)	6 (14.3%)	23 (15.2%)
Floor of mouth	64 (20.8%)	22 (19.6%)	7 (17.5%)	11 (26.2%)	28 (18.5%)
Mandible	77 (25.0%)	28 (25.0%)	13 (32.5%)	11 (26.2%)	39 (25.8%)
Maxilla + hard palate	39 (12.7%)	14 (12.5%)	5 (12.5%)	1 (2.4%)	16 (10.6%)
Cheek	24 (7.8%)	8 (7.1%)	3 (7.5%)	7 (16.7%)	17 (11.3%)
Soft palate	15 (4.9%)	7 (6.3%)	1 (2.5%)	0 (0.0%)	7 (4.6%)
Extraoral	44 (14.3%)	17 (15.2%)	4 (10.0%)	6 (14.3%)	21 (13.9%)
*Recipient vessel (n)*					
External carotid artery	16 (5.2%)	5 (4.5%)	1 (2.5%)	4 (9.5%)	8 (5.3%)
Facial artery	120 (39.0%)	41 (36.6%)	17 (42.5%)	20 (47.6%)	60 (39.7%)
Lingual artery	15 (4.9%)	4 (3.6%)	2 (5.0%)	2 (4.8%)	5 (3.3%)
Superior thyroid artery	147 (47.7%)	59 (52.7%)	20 (50.0%)	16 (38.1%)	75 (49.7%)
Other	10 (3.2%)	3 (2.7%)	0 (0.0%)	0 (0.0%)	3 (2.0%)
*Surgery duration* (min)	545.0 (155.0)	547.5 (158.0)	550.5 (140.0)	539.5 (182.0)	545.0 (153.0)
*Flap ischemia duration* (min)	105.5 (34.0)	109.0 (38.0)	99.0 (31.0)	101.5 (26.0)	107.0 (36.0)
*Smoking status (n)*					
No	182 (59.1%)	66 (58.9%)	27 (67.5%)	25 (59.5%)	93 (61.6%)
Yes	126 (40.9%)	46 (41.1%)	13 (32.5%)	17 (40.5%)	58 (38.4%)
*Prior neck dissection (n)*					
No	249 (80.8%)	97 (86.6%)	35 (87.5%)	35 (83.3%)	127 (84.1%)
Yes	59 (19.2%)	15 (13.4%)	5 (12.5%)	7 (16.7%)	24 (15.9%)
Prior neck irradiation (n)***					
No	277 (89.9%)	100 (89.3%)	34 (85.0%)	34 (81.0%)	132 (87.4%)
Yes	31 (10.1%)	12 (10.7%)	6 (15.0%)	8 (19.0%)	19 (12.6%)

Parameters are indicated as numbers (with percentage) for categorical data (sex, ASA, flap type, flap location, recipient vessel, smoking status, prior neck dissection, prior neck irradiation) or median (with interquartile range) for metric data (age, BMI, surgery duration, flap ischemia duration) (separately described for all patients (All), for patients with arterial hypertension (AHTN), for patients with diabetes mellitus (DM), for patients with atherosclerotic vascular disease (ASVD), and for patients with arterial hypertension, diabetes mellitus and or atherosclerotic vascular disease (AHTN/DM/ASVD)); testing for differences between groups with Chi-squared test (sex, ASA, flap type, smoking status, prior neck irradiation), Fisher’s exact test (prior neck dissection), Freeman–Halton test (flap location, recipient vessel) or Mann–Whitney test (age, BMI, surgery duration, flap ischemia duration): *p < 0.05 testing patients with AHTN vs. patients without AHTN; **p < 0.05 testing patients with DM vs. patients without DM; ***p < 0.05 testing patients with ASVD vs. patients without ASVD; ****p < 0.05 testing patients with AHTN/DM/ASVD vs. patients without AHTN/DM/ASVD

*AHTN* arterial hypertension, *DM* diabetes mellitus, *ASVD* atherosclerotic vascular disease, *BMI* body mass index, *ASA* American Society of Anesthesiologists score, *RFFF* radial free forearm flap, *ALTF* anterolateral thigh flap, *FFF* fibula free flap

### Comparison of flap perfusion parameters between groups

**Fig. 1:**
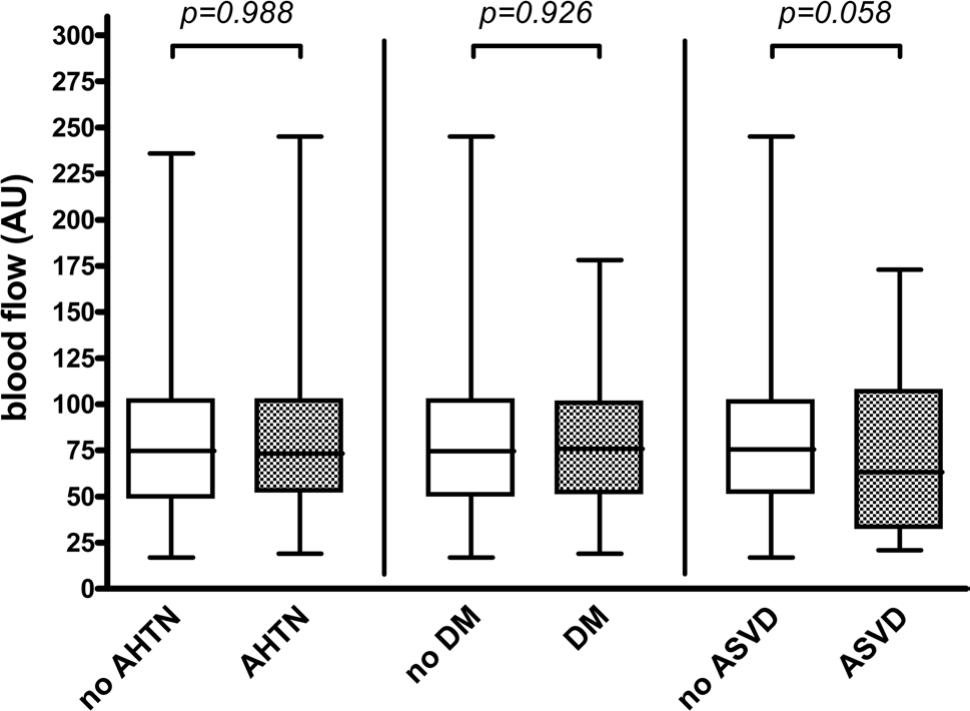
Intraoperative blood flow measurement. Box plot for blood flow (AU) for intraoperative measurement (separately described for patients without and with AHTN, for patients without and with DM, and for patients without and with ASVD); *p* values corresponding to testing for differences with Mann–Whitney test; *AHTN* arterial hypertension, *DM* diabetes mellitus, *ASVD* atherosclerotic vascular disease, *AU* arbitrary units. (Created with GraphPad Prism 4, GraphPad Software, San Diego, USA)

**Fig. 2:**
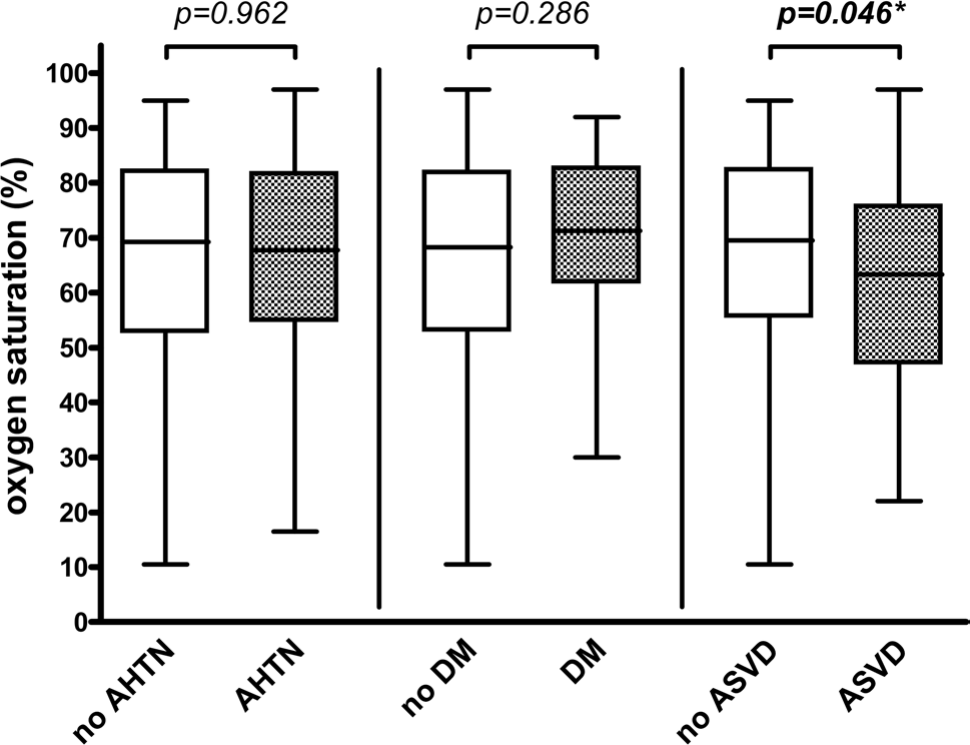
Intraoperative hemoglobin oxygen saturation measurement. Box plot for hemoglobin oxygen saturation (%) for intraoperative measurement (separately described for patients without and with AHTN, for patients without and with DM, and for patients without and with ASVD); *p* values corresponding to testing for differences with Mann–Whitney test; significant *p* values are bold (*p = 0.094 upon adjustment for sex, age, ASA (1 + 2 vs. 3 + 4), prior neck irradiation, flap type (RFFF vs. ALTF vs. FFF), flap location (intraoral vs. extraoral), mean arterial blood pressure (mmHg), and administered catecholamine dose (µg/min/kg) in multiple linear regression analysis); *AHTN* arterial hypertension, *DM* diabetes mellitus, *ASVD* atherosclerotic vascular disease. (Created with GraphPad Prism 4, GraphPad Software, San Diego, USA)

Intraoperative blood flow was similar between patients with and without AHTN, DM, or ASVD (*p* = 0.988, *p* = 0.926, and *p* = 0.058, respectively) (Table 2, Fig. 1). No differences were observed in intraoperative hemoglobin concentration between patients with and without AHTN, DM, or ASVD (*p* = 0.099, *p* = 0.694, and *p* = 0.258, respectively) (Table 2). Intraoperative hemoglobin oxygen saturation was similar in patients with and without AHTN or DM (*p* = 0.962 and *p* = 0.286, respectively) and lower in patients with ASVD than in patients without ASVD (63.3% vs. 69.5%, *p* = 0.046) (Table 2, Fig. 2). Upon adjustment for sex, age, ASA, prior neck irradiation, flap type, flap location, mean arterial blood pressure, and administered catecholamine dose, this difference did not persist (*p* = 0.094).

**Table 2:** Flap perfusion parameters of the study population

Variable	No	Yes	*p* value
Intraoperative measurement			
*AHTN*			
Blood flow (AU)	74.8 (52.9)	73.3 (50.0)	0.988
Hemoglobin concentration (AU)	53.5 (18.5)	51.5 (16.9)	0.099
Hemoglobin oxygen saturation (%)	69.3 (29.8)	67.8 (27.3)	0.962
*DM*			
Blood flow (AU)	74.5 (51.9)	76.0 (52.4)	0.926
Hemoglobin concentration (AU)	52.0 (19.9)	54.5 (17.1)	0.694
Hemoglobin oxygen saturation (%)	68.3 (29.0)	71.3 (21.3)	0.286
*ASVD*			
Blood flow (AU)	75.5 (49.4)	63.3 (73.9)	0.058
Hemoglobin concentration (AU)	52.8 (19.5)	51.5 (17.3)	0.258
Hemoglobin oxygen saturation (%)	69.5 (27.0)	63.3 (28.1)	0.046*
*AHTN/DM/ASVD*			
Blood flow (AU)	76.0 (51.8)	71.5 (53.3)	0.160
Hemoglobin concentration (AU)	53.5 (18.5)	52.0 (17.5)	0.244
Hemoglobin oxygen saturation (%)	70.0 (28.8)	68.0 (29.0)	0.691
Postoperative measurement			
*AHTN*			
Blood flow (AU)	81.0 (54.5)	85.3 (61.6)	0.924
Hemoglobin concentration (AU)	46.0 (19.9)	48.5 (17.9)	0.224
Hemoglobin oxygen saturation (%)	61.8 (26.4)	60.0 (27.3)	0.635
*DM*			
Blood flow (AU)	80.8 (57.1)	85.0 (52.8)	0.396
Hemoglobin concentration (AU)	47.5 (18.5)	43.5 (16.4)	0.028**
Hemoglobin oxygen saturation (%)	60.5 (26.5)	61.3 (27.1)	0.969
*ASVD*			
Blood flow (AU)	85.0 (55.3)	67.5 (54.0)	0.036***
Hemoglobin concentration (AU)	47.0 (19.1)	43.8 (18.6)	0.190
Hemoglobin oxygen saturation (%)	61.0 (26.1)	57.5 (28.5)	0.339
*AHTN/DM/ASVD*			
Blood flow (AU)	83.0 (54.5)	81.0 (58.0)	0.213
Hemoglobin concentration (AU)	47.0 (21.0)	46.5 (18.5)	0.751
Hemoglobin oxygen saturation (%)	62.0 (26.0)	60.5 (27.5)	0.682

Parameters are indicated as median (with interquartile range) for intraoperative and postoperative measurement (separately described for patients without and with AHTN, for patients without and with DM, for patients without and with ASVD, and for patients without and with arterial hypertension, diabetes mellitus and or atherosclerotic vascular disease (AHTN/DM/ASVD)); *p* values corresponding to testing for differences between groups with Mann–Whitney test; significant *p* values are bold (*p = 0.094 upon adjustment for sex, age, ASA (1 + 2 vs. 3 + 4), prior neck irradiation, flap type (RFFF vs. ALTF vs. FFF), flap location (intraoral vs. extraoral), mean arterial blood pressure (mmHg), and administered catecholamine dose (µg/min/kg) in multiple linear regression analysis; **p = 0.060 upon adjustment for age, BMI (≤ 25 vs. > 25), flap type (RFFF vs. ALTF vs. FFF), flap location (intraoral vs. extraoral), mean arterial blood pressure (mmHg), and administered catecholamine dose (µg/min per kg) in multiple linear regression analysis; ***p = 0.311 upon adjustment for sex, age, ASA (1 + 2 vs. 3 + 4), prior neck irradiation, flap type (RFFF vs. ALTF vs. FFF), flap location (intraoral vs. extraoral), mean arterial blood pressure (mmHg), and administered catecholamine dose (µg/min per kg) in multiple linear regression analysis

*AHTN* arterial hypertension, *DM* diabetes mellitus, *ASVD* atherosclerotic vascular disease, *AU* arbitrary units, *RFFF* radial free forearm flap, *ALTF* anterolateral thigh flap, *FFF* fibula free flap

**Fig. 3:**
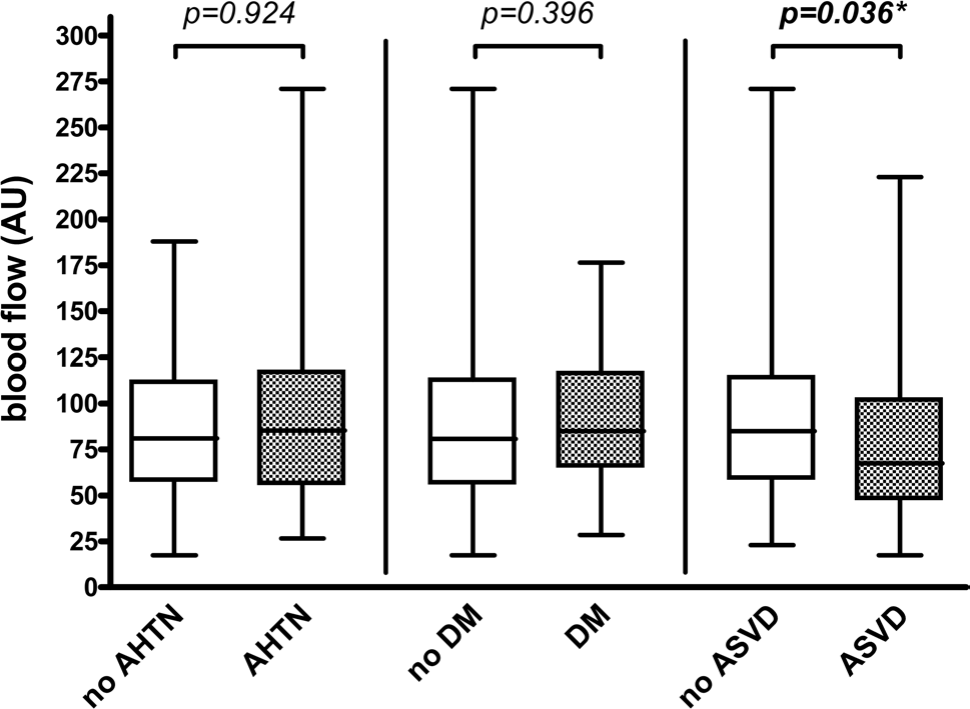
Postoperative blood flow measurement. Box plot for blood flow (AU) for postoperative measurement (separately described for patients without and with AHTN, for patients without and with DM, and for patients without and with ASVD); *p* values corresponding to testing for differences with Mann–Whitney test; significant *p* values are bold (*p = 0.311 upon adjustment for sex, age, ASA (1 + 2 vs. 3 + 4), prior neck irradiation, flap type (RFFF vs. ALTF vs. FFF), flap location (intraoral vs. extraoral), mean arterial blood pressure (mmHg), and administered catecholamine dose (µg/min per kg) in multiple linear regression analysis); *AHTN* arterial hypertension, *DM* diabetes mellitus, *ASVD* atherosclerotic vascular disease, *AU* arbitrary units. (Created with GraphPad Prism 4, GraphPad Software, San Diego, USA)

**Fig. 4:**
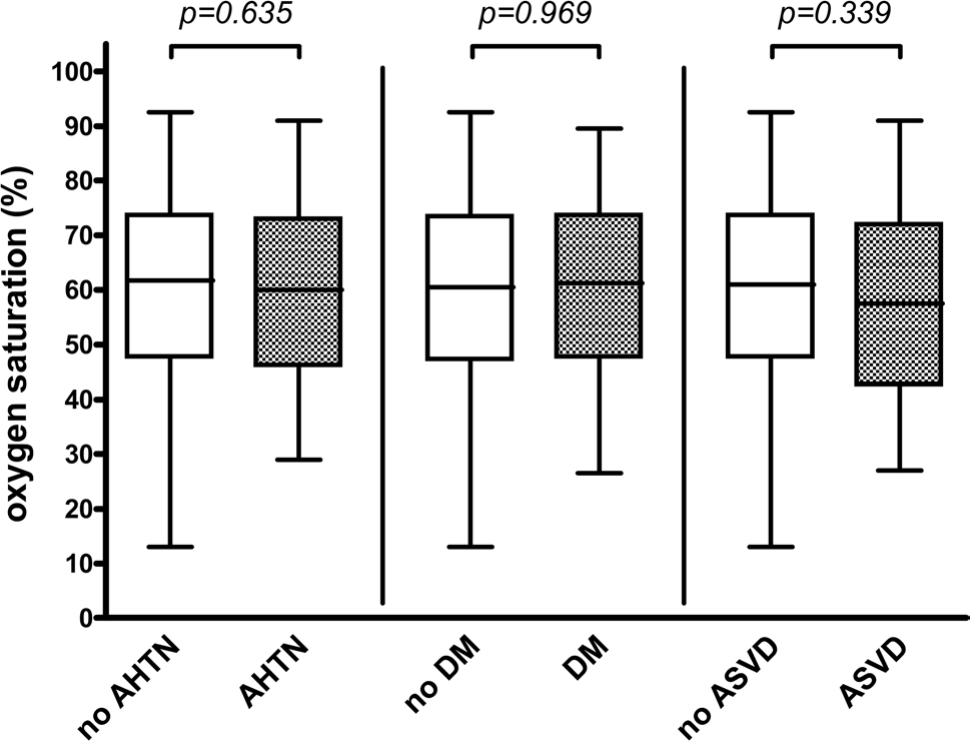
Postoperative hemoglobin oxygen saturation measurement. Box plot for hemoglobin oxygen saturation (%) for postoperative measurement (separately described for patients without and with AHTN, for patients without and with DM, and for patients without and with ASVD); *p* values corresponding to testing for differences with Mann–Whitney test; *AHTN* arterial hypertension, *DM* diabetes mellitus, *ASVD* atherosclerotic vascular disease. (Created with GraphPad Prism 4, GraphPad Software, San Diego, USA)

Postoperative blood flow was similar in patients with and without AHTN or DM (*p* = 0.924 and *p* = 0.396, respectively) and lower in patients with ASVD than in patients without ASVD (67.5 AU vs. 85.0 AU, *p* = 0.036) (Table 2, Fig. 3). Upon adjustment for sex, age, ASA, prior neck irradiation, flap type, flap location, mean arterial blood pressure, and administered catecholamine dose, this difference did not persist (*p* = 0.311). Postoperative hemoglobin concentration was similar in patients with and without AHTN or ASVD (*p* = 0.224 and *p* = 0.190, respectively) and lower in patients with DM than in patients without DM (43.5 AU vs. 47.5 AU, *p* = 0.028) (Table 2). This difference did not persist upon adjustment for age, BMI, flap type, flap location, mean arterial blood pressure, and administered catecholamine dose (*p* = 0.060). No differences were observed for postoperative hemoglobin oxygen saturation between patients with and without AHTN, DM, or ASVD (*p* = 0.635, *p* = 0.969, and *p* = 0.339, respectively) (Table 2, Fig. 4).

No differences were observed in all perfusion measurements between patients with and without AHTN, DM, and or ASVD (all *p* > 0.05) (Table 2).

## Discussion

This study investigated the impact of vascular comorbidities, i.e., AHTN, DM, and ASVD, on microvascular free flap perfusion, as these comorbidities are associated with impaired systemic macrovascular and microvascular perfusion and are common in patients undergoing head and neck reconstruction with microvascular free flaps [1–9]. Impaired systemic macrovascular and microvascular perfusion caused by vasoconstriction, vascular stiffness, and capillary scarcity in patients with AHTN and DM, and impaired systemic macrovascular perfusion caused by vascular plaque formation, wall thickening, and lumen obstruction in patients with ASVD, might also compromise free flap perfusion as a prerequisite for flap viability and survival, and thus for reconstruction success [5–12, 14, 19, 20]. However, microvascular free flaps have been successfully used in patients with AHTN, DM, or ASVD despite presumably compromised physical conditions [2–9, 15, 16].

Hence, this study aimed to investigate the impact of these vascular comorbidities on microvascular free flap perfusion with a view to providing a possible explanation for the successful use of microvascular free flaps despite presumably compromised physical conditions. This study did not examine the impacts of these comorbidities on flap success nor evaluate flap perfusion cut-off values to determine flap failure, as several previous studies have already addressed both aspects [2–4, 11, 13, 15, 16].

In this study, patients were confirmed to have AHTN or DM when their diagnoses were confirmed according to the disease-specific guidelines. Patients with diabetes mellitus other than type 2 were only a minority and were excluded to reduce heterogenicity. Patients with confirmed diagnoses of cerebrovascular disease, coronary heart disease, and/or peripheral arterial disease were grouped together as patients with ASVD since these diseases are predominantly of atherosclerotic origin [21, 22]. In the multivariable analysis, adjustment was made for factors with observed differences between groups (e.g., sex, age, BMI, ASA and prior neck irradiation) and for factors with presumed impact on flap perfusion (e.g., flap type, mean arterial blood pressure, and catecholamine dose administered) [13, 18, 23].

The results of this study demonstrated that flap perfusion parameters, i.e., flap blood flow, hemoglobin concentration and hemoglobin oxygen saturation, were similar between patients with and without AHTN, DM, and ASVD, both intraoperatively (immediately after anastomosis release) and postoperatively (on the first postoperative morning). This suggests that these comorbidities have no impacts on the perfusion of microvascular free flaps used for reconstruction of the head and neck region. With regard for a comparable study that also measured microvascular free flap perfusion with the O2C device, the measured values for postoperative flap perfusion in this study were corresponding [24]. However, in univariable testing, some differences were found between groups. The lower intraoperative hemoglobin oxygen saturation in patients with ASVD compared to patients without ASVD in the univariable analysis may be attributed to the tendentially lower blood flow of patients with ASVD, since hemoglobin oxygen saturation depends on blood flow [11]. The lower postoperative hemoglobin concentration in patients with DM compared to patients without DM may reflect the capillary scarcity associated with DM, as hemoglobin is primarily located in the capillary-venous compartment of the microcirculation [11]. The tendentially lower intraoperative and significantly lower postoperative blood flow in patients with ASVD compared to patients without ASVD may be related to a lower adaptability of their flaps to the perfusion conditions at the cervical recipient sites in terms of vasodilatation (caused, for example, by ASVD-related vascular stiffness), as microvascular free flaps usually show an increase in blood flow after anastomosis to the cervical recipient vessel [5, 13]. Interestingly, in the present study, the commonly observed increase in flap blood flow between the intraoperative and postoperative perfusion measurements was relatively lowest in patients with ASVD (6.6% vs. 8.3–16.4%). However, neither difference persisted in the multivariable analysis, suggesting that differences were likely due to factors other than the presence of AHTN, DM, or ASVD. The comparable flap perfusion in patients with and without AHTN, DM, and ASVD may be explained by the absence—or, at least, insignificance—of presumably compromised physical conditions in the recipient and donor site vasculature and flap tissues [25].

With regard to the limitations of the current study, several aspects should be mentioned. Although the O2C device has been shown to be a reliable method for measuring flap perfusion, a moist environment due to salivatory flow and wound secretion as well as differences in skin temperature may influence the measurement [10, 18, 24, 26, 27]. However, all skin surfaces were dried prior to measurement, an adjustment for flap location (intraoral vs. extraoral) was made in the multivariable testing in view of potentially higher measurement difficulties with intraoral flaps due to the moist environment, and all measurements were performed in rooms (operating theater and intensive care unit) with room climate control, all which might mitigate these potential influences. Nevertheless, potential differences in several factors that might affect flap perfusion (e.g., the length and diameter of the pedicle vessel) among patients cannot be excluded. In addition, it cannot be precluded that groups were heterogenous with respect to the extent of vascular changes caused by AHTN and DM (e.g., owing to differences in disease duration). Moreover, the group of patients with ASVD included individuals with different diseases. Unfortunately, preoperative and postoperative reference values for flap perfusion before flap harvest and for the opposite donor site, respectively, were not available, and only smokers (current and former) and non-smokers could be distinguished due to the lack of information regarding the timepoints of smoking cessation. Notably, the success of microvascular free flaps depends on multiple factors (e.g., the technical requirements for anastomosis) and flap perfusion is only one contributing factor [14, 28]. Moreover, flap perfusion is a critical factor that affects flap viability and flap survival well beyond the short time frame examined in this study, and it cannot be ruled out that flap perfusion may change over time beyond the period investigated [14, 28].

This study demonstrated that vascular comorbidities such as AHTN, DM, and ASVD have no impact on initial microvascular free flap perfusion. Interestingly, in our data set, before exclusion of patients with flap failure, no difference was observed between patients with and without AHTN, DM or ASVD in terms of flap failure (1 (0.9%) vs. 4 (2.0%), Fisher’s exact test *p* = 0.657; 0 (0.0%) vs. 5 (1.8%), Fisher’s exact test *p* = 1.000; 2 (4.5%) vs. 3 (1.1%), Fisher’s exact test *p* = 0.146). Although the success of microvascular free flaps depends on multiple factors, unrestricted flap perfusion in patients with AHTN, DM, or ASVD may reflect both initial functionality of the technical procedure as well as an equivalent basis for the further course and thus contribute to the success of microvascular free flaps used for head and neck reconstruction in these patients [1–4, 11, 12, 14, 28].

## Conclusion

In microvascular free flaps used for head and neck reconstruction, flap perfusion parameters (e.g., flap blood flow and hemoglobin oxygen saturation) did not differ between patients with and without AHTN, DM, and ASVD. These results indicate that these comorbidities have no initial impact on flap perfusion. This may contribute to the successful use of microvascular free flaps for reconstruction in the head and neck region in patients with AHTN, DM, and ASVD.

## Data Availability

The datasets generated and analyzed during the current study are available from the corresponding author on reasonable request.
